# Trimethylamine N-oxide, a gut microbiota-dependent metabolite of choline, is positively associated with the risk of primary liver cancer: a case-control study

**DOI:** 10.1186/s12986-018-0319-2

**Published:** 2018-11-20

**Authors:** Zhao-Yan Liu, Xu-Ying Tan, Qi-Jiong Li, Gong-Cheng Liao, Ai-Ping Fang, Dao-Ming Zhang, Pei-Yan Chen, Xiao-Yan Wang, Yun Luo, Jing-An Long, Rong-Huan Zhong, Hui-Lian Zhu

**Affiliations:** 1 Guangdong Provincial Key Laboratory of Food, Nutrition and Health, School of Public Health, Sun Yat-sen University, Guangzhou, 510080 People’s Republic of China; 2 Department of Hepatobiliary Oncology, Sun Yat-sen University Cancer Center, Guangzhou, 510060 People’s Republic of China

**Keywords:** Trimethylamine N-oxide (TMAO), Choline, Gut microbiota metabolite, Liver cancer, Case control study

## Abstract

**Background:**

Evidence has suggested a potential link exists between trimethylamine-N-oxide (TMAO), a choline-derived metabolite produced by gut microbiota, and some cancers, but little is known for primary liver cancer (PLC).

**Methods:**

A case-control study was designed including 671 newly diagnosed PLC patients and 671 control subjects frequency-matched by age (±5 years) and sex, in Guangdong province, China. High-performance liquid chromatography with online electrospray ionization tandem mass spectrometry (HPLC-MS/MS) was used to measure serum TMAO and choline. The associations between these biomarkers and PLC risk were evaluated using logistic regression models.

**Results:**

Serum TMAO concentrations were greater in the PLC group than the control group (*P* = 0.002). Logistic regression analysis showed that the sex- and age-adjusted odds ratio (OR) and (95% confidence interval [CI]) was 3.43 (2.42–4.86) when comparing the top and bottom quartiles (Q4 vs Q1). After further adjusting for more selected confounders, the OR (95% CI) remained significant but was attenuated to 2.85 (1.59–5.11) (Q4 vs Q1). The multivariable-adjusted ORs (95% CIs) across quartiles of choline were 0.35–0.15 (*P*_-trend_ < 0.001).

**Conclusion:**

Higher serum levels of TMAO were associated with increased PLC risk. The association was stronger in those with lower serum levels of choline. Additional large prospective studies are required to confirm these findings.

**Trial registration:**

This study was registered at clinicaltrials.gov as NCT 03297255.

## Background

Primary liver cancer (PLC) is one of the deadliest malignant tumors worldwide [1]. An estimated 0.8 million new diagnoses of liver cancer and deaths occurred worldwide during 2012, with approximately 50% of the total number occurring in China alone [2]. Chronic viral hepatitis B (HBV) and C (HCV) and exposure to aflatoxin are the predominant risk factors for PLC [3]. Since the implementation of the routine infant HBV immunization [4], as well as improvements in hygiene and sanitation [5], infection-related liver cancer rates are decreasing in historically high-risk areas [5]. However, non-infection-related risk factors for PLC such as alcohol abuse, tobacco smoking, non-alcoholic fatty liver disease (NAFLD), obesity, and type 2 diabetes, have raised concern over recent years [5]. In addition, growing evidence suggests that diet plays a crucial role [6–8] in PLC development.

Choline is an essential nutrient in one-carbon metabolism [8], its effect on the level of DNA methylation is postulated to play an important role in tumor development [8–10]. Previous studies have shown that a deficiency in dietary choline may promote the development of liver cancer both in animals and humans [11, 12]. Additionally, circulating choline was also found to be associated with liver cancer risk in a nested case-control study including 297 male liver cancer patients and 631 male matched controls [13].

Trimethylamine N-oxide (TMAO) is a gut microbiota-dependent metabolite of choline that is formed in the liver by the hepatic enzyme flavin-containing monooxygenase-3 (FMO3) [14]. With the development of microbial metabolomics, investigators have demonstrated TMAO as a risk factor for many diseases [15] including cardiovascular disease [14, 16, 17], type 2 diabetes [18, 19] and chronic kidney disease [20]. All of these diseases may increase the risk of PLC [21, 22]. Moreover, some recent studies suggest a link between TMAO and the risk of cancer development, especially colorectal cancer (CRC) [23–25]. Sajin Bae et al. first reported that plasma TMAO was positively associated with rectal cancer in a nested case-control study including 835 matched case-control pairs [23]. Another study revealed a strong genetic link between CRC and TMAO using genome-wide systems analysis to construct chemical-gene, disease-gene, and protein-protein interaction data from multiple large-scale data resources [25]. Furthermore, through the use of systematic disease enrichment analysis, the study also demonstrated that TMAO may be related to other types of cancer, including liver cancer [25]. Since the generation of TMAO involves crosstalk between the gut and liver (dietary choline/betaine/L-carnitine → trimethylamine formed in gut→ TMAO formed in liver) [17], the link between TMAO and CRC suggests that TMAO may also be related to PLC. Although none of the existing studies have assessed the association between TMAO and liver cancer, our previous research demonstrated a significant positive association between serum TMAO and NAFLD [26]. Since NAFLD is a well-established risk factor for PLC, the association supports a possible link between TMAO and PLC.

Collectively, we conducted a case-control study to investigate the association between serum TMAO, a gut microbiota-dependent metabolite of choline, and PLC risk in a large Chinese population.

## Methods

### Study population

A cross-sectional case-control study was conducted. Recruitment methods for PLC patients have been previously detailed. [6] Newly diagnosed (within one month) adults with PLC from Sun Yat-sen University Cancer Center in the Guangdong province were consecutively enrolled between September 2013 and April 2017. All cases were diagnosed according to the National Comprehensive Cancer Network (NCCN) Clinical Practice Guidelines in Oncology: Hepatobiliary Cancers [27]. PLC patients were excluded if they (1) had no blood samples, (2) had a history of other cancers, or (3) had a history of stroke or chronic kidney disease. Controls meeting the same inclusion and exclusion criteria, with the exception of liver cancer, were concurrently enrolled from local communities in the Guangdong province. A total of 671 eligible PLC patients and 671 controls, frequency-matched by age (±5 years) and sex, were included in the present analyses. Written informed consent was provided by all study participants, and the study protocol was approved by the Ethics Committee of the School of Public Health at Sun Yat-sen University.

### Data collection

Information on socio-demographic characteristics and lifestyle habits over the past year was obtained by well-trained research interviewers using a structured questionnaire. Household income was divided into three groups: ≤2000, 2001–6000, and > 6000 Yuan/month/person. Occupation was determined by labor intensity. Participants’ marital status was determined as either married or not married. Education was grouped into three levels: primary school or below, secondary and high school, and college or above. Participants who smoked at least one cigarette per day or drank alcohol at least once a week continuously for at least six months in one’s whole life, were defined as smokers or alcohol drinkers.

Anthropometric data including waist circumference (WC), height (m), and weight (kg) were obtained using standard procedures and measuring equipment. Body mass index (BMI; kg/m^2^) was calculated. Blood pressure (BP) was measured using a calibrated sphygmomanometer (Hawksley, WA Baum Co, USA). Serostatus of hepatitis B surface antigen was determined by enzyme linked immunosorbent assay. Serum total cholesterol (TC) and triglycerides (TG) were analyzed using enzymatic colorimetric tests; the elective inhibition method was used to measure serum high-density lipoprotein cholesterol (HDL-C). Fasting blood glucose (FBG) was determined using the hexokinase method. All biochemical parameters were determined using commercially available kits on an automatic biochemistry analyzer (Advia1650 Autoanalyzer, Byer Diagnostics, Leverkusen, Germany). Metabolic syndrome (Mets) was diagnosed based on WC, BP, TG, HDL-C and FBG, according to International Diabetes Federation criteria [28].

### Laboratory analysis of serum TMAO and choline

Fasting serum samples were isolated and stored at − 80 °C until analysis. Serum TMAO and choline were assessed using high-performance liquid chromatography with online electrospray ionization tandem mass spectrometry (HPLC-MS/MS) (Agilent 6400 Series Triple Quad LCMS; CA, USA) [29], using multi-reaction monitoring (MRM) functions. 100 μl of acetonitrile containing 10 μM of internal standards [d9-TMAO (Toronto Research Chemicals Inc., Toronto, Canada), d9-choline (Sigma-Aldrich, St. Louis, USA)] was added to 60 μl of either the serum sample or standards. The samples were then centrifuged at 13,000×g for 10 min to precipitate the proteins. Finally, the remaining supernatant was injected into a normal-phase silica column (2.1 mm × 100 mm, 5 μM) and equilibrated with 30% solution A (15 mmol/L ammonium formate in water, pH 3.0) and 70% solution B (acetonitrile) under isocratic elution with a flow rate of 0.2 mL/min. Ten pairs of duplicate control samples were randomly interspersed to assess laboratory precision. The coefficients of variation for the between-run assays were 6.0 and 4.9% for TMAO and choline, respectively.

### Statistical analysis

Data were analyzed using SPSS version 20.0 for Windows (SPSS Inc., Chicago, IL, USA). *P*-values were based on two-tailed tests and *P* < 0.05 was considered to be statistically significant.

All analyses included men and women combined, with the exception of analyses stratified by sex. Differences in socio-demographic characteristics, presence of Mets, serum TMAO, and choline concentrations between patients and controls were compared by t-test, chi-squared test, and Wilcoxon rank-sum test as appropriate. Serum TMAO and choline concentrations were grouped into quartiles (Q1–Q4) based on control subjects, and then the cutoffs were applied to the PLC patients. Logistic regression models were used to calculate odds ratios (ORs) and corresponding 95% confidence intervals (CIs) with the first quartile as the reference. The forward stepwise method was used to select variables included in the multivariable analyses, and only those that were statistically significant (*P* < 0.10) were included in the final models. Only age and sex were adjusted for in model 1. Further adjustments were made for BMI, household income, occupation, marital status, smoking status, alcohol use, presence of Mets, and HBV infection status in model 2. Linear trends across increasing quartiles were assessed by assigning quartiles as continuous variables in the regression models. To investigate possible associations between various joint effects of serum TMAO and choline with PLC risk, four groups were studied according to combinations of serum TMAO and choline that were stratified based on the study population medians. The group with low-TMAO and high-choline was considered as the reference group (group 1).

In stratified analyses, we divided serum TMAO and choline into quartiles and then combined the second and third quartiles into a middle 50% to examine whether associations between serum TMAO/choline and PLC risk were different in various subgroups (HBV-infected subjects vs non-HBV-infected subjects, alcohol drinkers vs non-alcohol drinkers, smokers vs non-smokers, men vs women, with Mets vs without Mets). Interactions were estimated via multiplicative interaction terms in the multivariate model 2.

## Results

### Basic characteristics

Basic characteristics of the 671 PLC patients and control pairs (569 male pairs and 102 female pairs) are presented in Table 1. The mean (±S.D.) age of PLC patients and non-PLC controls were 58.6 ± 7.6 years and 58.6 ± 7.5 years, respectively. Compared with controls, PLC patients were more likely to have a lower BMI, lower level of education, lower incidence of Mets, and higher household income. A significantly greater proportion of PLC patients had heavy intensity occupations, were married, were alcohol drinkers, and were infected with HBV (*P* < 0.05 for all variables). No significant differences were observed in smoking status between patients and controls.

**Table 1:** Comparison of selected characteristics between PLC cases and controls

Variables	PLC	Controls	*P* value
Age (years)^a^	58.6 ± 7.6	58.6 ± 7.5	0.981
Men, *n* (%)	569 (84.8)	569 (84.8)	–
BMI (kg/m^2^) ^a^	22.8 ± 3.1	23.8 ± 3.1	< 0.001
Household income (Yuan/month/person), *n* (%)			
≤2000	206 (30.7)	355 (52.9)	< 0.001
2001–6000	410 (61.1)	283 (42.2)	
> 6000	55 (8.2)	33 (4.9)	
Occupation, *n* (%)			
Light intensity of activity	246 (36.7)	365 (54.4)	< 0.001
Moderate intensity of activity	209 (31.2)	193 (28.8)	
Heavy intensity of activity	216 (32.2)	113 (16.8)	
Married, *n* (%)	658 (98.1)	643 (95.8)	0.017
Education level, *n* (%)			
Primary school or below	176 (26.2)	38 (5.7)	< 0.001
Secondary & High school	387 (57.7)	409 (61.0)	
College or above	108 (16.1)	224 (33.4)	
Smoker, *n* (%)	302 (45.0)	272 (40.6)	0.098
Alcohol drinker, *n* (%)	186 (27.7)	85 (12.7)	< 0.001
HBV infection, n (%)	582 (86.7)	60 (8.9)	< 0.001
Mets, *n* (%)	128 (19.1)	174 (25.9)	0.003
Serum choline (μmol/L) ^a^	12.88 ± 4.53	17.33 ± 5.15	< 0.001
Serum TMAO (μmol/L) ^b^	2.07 (1.32,3.49)	1.61 (0.87,2.92)	0.002

Abbreviations: *BMI* = body mass index, *PLC* = primary liver cancer, *HBV* = hepatitis B virus, *Mets* = Metabolic syndrome, *TMAO* = trimethylamine N-oxide

^a^Continuous values are mean ± S.D.

^b^Value is median (IQR)

Compared with controls, PLC patients had significantly higher serum levels of TMAO (2.07 (1.32, 3.49) μmol/L vs 1.61 (0.87, 2.92) μmol/L)) and lower levels of choline (12.88 ± 4.53 μmol/L vs 17.33 ± 5.15 μmol/L). All *P-*values were < 0.05.

### Associations between serum TMAO, choline, and PLC risk

Associations between serum TMAO, choline and PLC risk are shown in Table 2. In the sex- and age-adjusted analysis, participants with higher serum TMAO had a significantly increased risk of PLC (*P*_-trend_ < 0.001); the OR (95% CI) was 3.43 (2.42–4.86) when comparing the top and bottom quartiles (Q4 vs Q1). After further adjusting for BMI, household income, occupation, marital status, smoking status, alcohol use, presence of Mets, and HBV infection status, the OR remained significant (*P*_-trend_ = 0.003) but was attenuated to 2.85. An inverse association between serum choline and PLC risk was found. A higher serum concentration of choline was associated with a significantly lower risk of PLC (*P*_-trend_ < 0.001). The adjusted OR (95% CI) for Q4 vs Q1 was 0.15 (0.08–0.28) in the multivariate-adjusted model 2.

**Table 2:** Odds ratios (ORs) and 95% confidence intervals (CIs) of PLC according to quartiles of serum TMAO and choline levels among controls

	Level (μmol/L)	n (Cases/Controls)	OR1 (95% CI) ^a^	OR2 (95% CI) ^b^
Serum TMAO				
Q1	≤0.87	66/168	1.00	1.00
Q2	0.87–1.61	167/168	2.53 (1.77–3.61)	2.41 (1.32–4.40)
Q3	1.61–2.92	213/168	3.23 (2.28–4.58)	2.91 (1.62–5.23)
Q4	≥2.92	225/167	3.43 (2.42–4.86)	2.85 (1.59–5.11)
*P*_-trend_			< 0.001	0.003
Serum choline				
Q1	≤13.27	425/167	1.00	1.00
Q2	13.27–16.95	134/168	0.29 (0.22–0.39)	0.35 (0.21–0.57)
Q3	16.95–21.03	68/168	0.15 (0.11–0.21)	0.22 (0.12–0.38)
Q4	≥21.03	44/168	0.09 (0.06–0.14)	0.15 (0.08–0.28)
*P*_-trend_			< 0.001	< 0.001

^a^Adjusted for age and sex

^b^Further adjusted for BMI, household income, occupation, marital status, smoking status, alcohol use, presence of metabolic syndrome, and HBV infection status

**Fig. 1:**
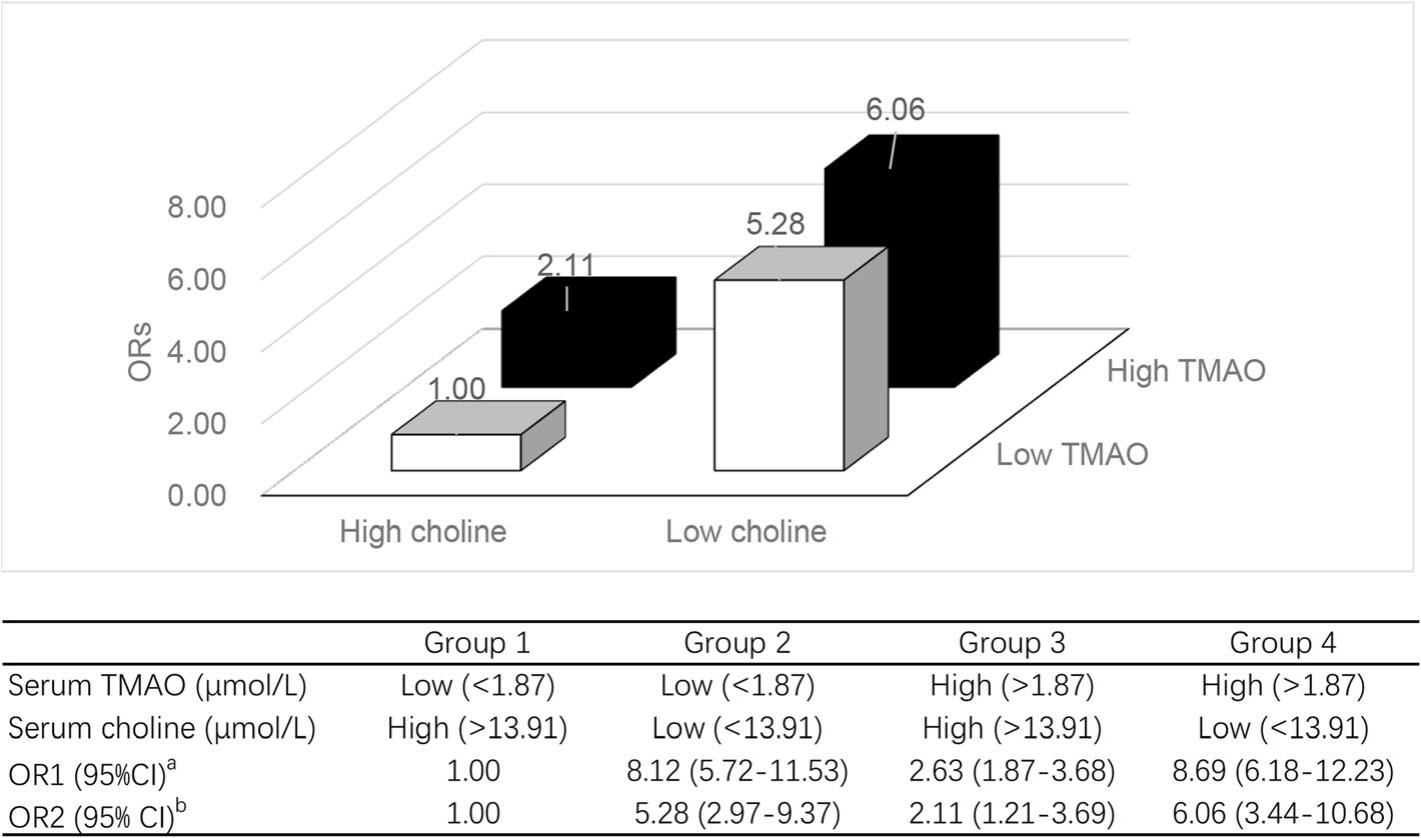
ORs and 95% CIs of PLC in four groups: low TMAO/high choline, low TMAO/low choline, high TMAO/high choline, and high TMAO/low choline. Groups were stratified based on the median TMAO and choline concentrations of the entire study population. ^a^Adjusted for age and sex; ^b^Further adjusted for BMI, household income, occupation, marital status, smoking status, alcohol use, presence of metabolic syndrome, and HBV infection status

In the four combined groups analyses, median concentrations of the whole study population were 1.87 μmol/L and 13.91 μmol/L for TMAO and choline, respectively. Compared with group 1 (low-TMAO and high-choline), participants with unfavorable factors (high-TMAO, low-choline, or both) all had an increased risk of PLC, those with high-TMAO and low-choline had the highest risk of PLC, the multivariate-adjusted OR (95% CI) for group 4 vs group 1 was 6.06 (3.44–10.68). Data are shown in Fig. 1.

### Stratified analyses

In risk analyses stratified by HBV infection status, alcohol use, smoking, sex, and presence of Mets, positive associations between TMAO and PLC risk were found only among non-HBV-infected patients (*P*_-trend_ = 0.006), non-drinkers (*P*_-trend_ = 0.001), non-smokers (*P*_-trend_ = 0.001) and participants without Mets (*P*_-trend_ = 0.006), but not among HBV-infected patients (*P*_-trend_ = 0.104), alcohol drinkers (*P*_-trend_ = 0.092), smokers (*P*_-trend_ = 0.418) and participants with Mets (*P*_-trend_ = 0.095). However, the *P*_-interaction_ was only significant between smokers and non-smokers (*P*_-interaction_ = 0.024).

The inverse associations of choline and PLC risk were not significantly modified, although stronger among non-smokers than smokers (*P*_-trend_ < 0.001, *P*_-interaction_ = 0.001), and stronger among women than men (*P*_-trend_ < 0.001, *P*_-interaction_ = 0.008). All of the stratified analyses are shown in Table 3.

**Table 3:** Associations between serum TMAO and choline levels with PLC risk stratified by selected factors

		Q1	Combined middle half	Q4	*P*_-trend_	*P*_-interaction_
Serum TMAO						
HBV infection^b^						
Yes	n (Cases/Controls)	76/15	333/30	173/15		0.095
	OR (95% CI) ^a^	1.00	1.83 (0.89–3.78)	2.07 (0.91–4.74)	0.104	
No	n (Cases/Controls)	8/152	53/306	28/153		
	OR (95% CI) ^a^	1.00	3.82 (1.63–8.94)	3.73 (1.50–9.30)	0.006	
Alcohol using^b^						
Yes	n (Cases/Controls)	13/21	121/43	52/21		0.777
	OR (95% CI) ^a^	1.00	2.54 (0.74–8.68)	3.44 (0.88–13.54)	0.092	
No	n (Cases/Controls)	51/145	274/295	160/146		
	OR (95% CI) ^a^	1.00	3.10 (1.69–5.67)	3.57 (1.83–6.96)	0.001	
Smoking^b^						
Yes	n (Cases/Controls)	41/68	175/136	86/68		0.024
	OR (95% CI) ^a^	1.00	1.32 (0.59–2.94)	1.47 (0.60–3.60)	0.418	
No	n (Cases/Controls)	30/99	209/200	130/100		
	OR (95% CI) ^a^	1.00	3.92 (1.84–8.35)	4.88 (2.13–11.19)	0.001	
Sex^b^						
Men	n (Cases/Controls)	74/140	312/287	183/142		0.166
	OR (95% CI) ^a^	1.00	2.04 (1.16–3.60)	2.28 (1.22–4.26)	0.016	
Women	n (Cases/Controls)	7/25	52/52	43/25		
	OR (95% CI) ^a^	1.00	1.79 (0.38–8.30)	5.23 (1.05–26.10)	0.021	
Metabolic syndrome^b^						
Yes	n (Cases/Controls)	14/43	75/88	39/43		0.473
	OR (95% CI) ^a^	1.00	3.04 (0.92–10.01)	3.45 (0.94–12.65)	0.095	
No	n (Cases/Controls)	50/124	307/249	186/124		
	OR (95% CI) ^a^	1.00	2.50 (1.35–4.62)	2.83 (1.44–5.59)	0.006	
Serum Choline						
HBV infection^b^						
Yes	n (Cases/Controls)	368/15	151/30	63/15		0.121
	OR (95% CI) ^a^	1.00	0.22 (0.11–0.46)	0.22 (0.09–0.50)	< 0.001	
No	n (Cases/Controls)	52/152	33/306	4/153		
	OR (95% CI) ^a^	1.00	0.24 (0.14–0.43)	0.05 (0.02–0.16)	< 0.001	
Alcohol using^b^						
Yes	n (Cases/Controls)	137/21	36/43	13/21		0.145
	OR (95% CI) ^a^	1.00	0.13 (0.05–0.37)	0.21 (0.05–0.90)	0.001	
No	n (Cases/Controls)	304/146	154/294	27/146		
	OR (95% CI) ^a^	1.00	0.34 (0.21–0.56)	0.13 (0.06–0.27)	< 0.001	
Smoking^b^						
Yes	n (Cases/Controls)	200/68	69/136	33/68		0.001
	OR (95% CI) ^a^	1.00	0.16 (0.08–0.34)	0.21 (0.08–0.53)	< 0.001	
No	n (Cases/Controls)	228/98	123/201	18/100		
	OR (95% CI) ^a^	1.00	0.37 (0.21–0.64)	0.12 (0.05–0.27)	< 0.001	
Sex^b^						
Men	n (Cases/Controls)	361/141	161/286	47/142		0.008
	OR (95% CI) ^a^	1.00	0.23 (0.14–0.38)	0.20 (0.10–0.38)	< 0.001	
Women	n (Cases/Controls)	69/25	30/52	3/25		
	OR (95% CI) ^a^	1.00	0.34 (0.11–1.10)	0.01 (0.00–0.08)	< 0.001	
Metabolic syndrome^b^						
Yes	n (Cases/Controls)	94/43	31/88	3/43		0.050
	OR (95% CI) ^a^	1.00	0.09 (0.03–0.26)	0.01 (0.00–0.10)	< 0.001	
No	n (Cases/Controls)	329/124	169/249	45/124		
	OR (95% CI)^a^	1.00	0.32 (0.19–0.53)	0.20 (0.10–0.40)	< 0.001	

^a^Adjusted for age, sex, BMI, household income, occupation, marital status, smoking status, alcohol use, presence of metabolic syndrome, and HBV infection status

^b^Stratified factors were not included in the corresponding model

## Discussion

### Key findings

To the best of our knowledge, the present study is the first to report on associations between serum TMAO and PLC risk in humans. We found that serum TMAO, a gut flora metabolite of choline, was positively associated with the development of PLC, whereas its precursor choline was inversely associated with PLC risk.

### TMAO and PLC

In recent years, many studies have reported associations between TMAO and chronic diseases. In both animals and humans, harmful effects of TMAO have been related to cardiovascular diseases (atherosclerosis [16, 17], thrombosis [30], hypertension [31]), chronic kidney disease [20], and NAFLD [26]. The contributions of TMAO in the development of these chronic diseases have brought attention to its potential role in carcinogenesis, as these diseases are all cancer risk factors. However, existing literature on associations between circulating TMAO and cancer is limited, and results have been inconclusive. Significant positive associations between TMAO and colorectal cancer were first reported in the Women’s Health Initiative Observational Study [23]. Liu et al. suggested a possible prognostic value of preoperative serum TMAO level in 108 colorectal cancer patients [24]. Additionally, Oellgaard et al. reported TMAO as a promising potential therapeutic target for gastrointestinal cancer [32]. Interestingly, divergent results were reported in male participants in the alpha-tocopherol and beta-carotene study, in which the association between TMAO and prostate cancer was positive [33], but null between TMAO and colorectal cancer [34].

Existing data on TMAO in relation to PLC risk is sparse. To our knowledge, the present study is the first to report on associations between serum TMAO and PLC risk in humans. Consistent with most of the previous findings, the present study found a significant association between serum TMAO and risk of PLC. We hypothesize two possible mechanisms. First, TMAO may contribute to liver injury by decreasing the total bile acid pool size and affecting hepatic TG levels [16], which may lead to the development of PLC. Second, TMAO may be an indirect risk factor by participating in the etiology of several chronic diseases, including atherosclerosis [16, 17], thrombosis [30], hypertension [31] and chronic kidney disease [20], which are all potential risk factors for PLC.

However, it is hard to determine from current literature whether serum TMAO is a risk factor or rather a biomarker for PLC status. Increasing evidence suggests the bacterial microbiome plays a key role in promoting liver cancer through the intestinal microbiota–liver axis [35]. Given that TMAO is a gut flora-dependent metabolite, elevated circulating TMAO concentrations may simply be a biomarker of gut microbiota composition fluctuation in PLC patients. Limited by the case-control design, this study could not definitively conclude whether elevated serum TMAO was involved in liver carcinogenesis, or if liver cancer caused the elevation in serum TMAO. Rong Xu et al. previously revealed a link between TMAO and colorectal cancer using a genome-wide systems analysis, and suggested a potential genetic link with other cancers [25]. These connections could be used in future studies of TMAO to better understand its role in PLC.

In the stratified analysis, we noted that the positive association between serum TMAO and PLC risk became insignificant among the HBV-infected patients, alcohol drinkers, smokers and participants with Mets, although the *P*_-interaction_ was only significant between smokers and non-smokers. A possible explanation would be that HBV, alcohol consumption, smoking and presence of Mets induced changes in gut flora composition and normal function [36–38], which consequently affected the generation of TMAO. In addition, HBV infection, alcohol drinking, smoking and presence of Mets are well-established PLC risk factors [5], their effects on PLC were much greater than TMAO, which may cover the risk effect of TMAO on PLC.

### Choline and PLC

The present study showed a robustly favorable relationship between serum choline and PLC. This was consistent with several previous studies [39–43] and with our previous case-control study, which reported an inverse association between dietary choline intake and PLC risk [11]. To date, only one case-control study, including 297 male HCC patients and 631 male matched controls, has reported on the relationship between serum choline and HCC risk [13], in which the favorable role of choline was also found. There are at least two potential mechanisms to explain the inverse associations between high serum choline and decreased PLC risk. First, choline is an important methyl donor in one-carbon metabolism, which can affect DNA methylation levels. Methyl donor deficiency could impair DNA methylation and further induce liver carcinogenesis [8, 44]. Animal models fed a choline-deficient diet have demonstrated an increased risk of liver cancer [44, 45]. Second, choline is necessary for normal lipid and TG transport from the liver, while defective VLDL secretion and fat accumulation may cause chronic liver diseases such as NAFLD or liver cancer [26].

In this study, we noted that serum TMAO and its precursor choline had opposite associations with PLC risk. One of the most likely reasons is that choline is not the only precursor of TMAO. For example, L-carnitine, a nutrient rich in red meat, could also be metabolized to TMAO [16]. In our previous study investigating the association of dietary choline intake and PLC risk, we found that red meat was an important source of dietary choline [11], which implied high dietary L-carnitine intake of the participants.

### Strengths and limitations

The present study had several strengths. First, this is the first study to report on associations between serum TMAO and PLC risk in humans. Moreover, the sample size (671 PLC patients and 671 matched controls) is relatively large, compared with the only existing serum choline and HCC risk case-control study (297 HCC patients and 631 matched controls) [13]. Second, only newly diagnosed PLC patients were included with comparable age- and sex-matched to minimize recall bias. Third, multiple confounding factors, including well-established risk factors for PLC, (i.e., HBV infection, alcohol use, smoking) were included in the analyses to reduce residual confounding.

However, several limitations warrant consideration. First, the blood samples were collected at the time point when the participants were diagnosed, a single measurement may not fully reflect the body’s long-term concentrations of choline or its metabolite TMAO. We cannot exclude the possibility that the development of PLC may itself affect concentrations of serum choline and TMAO, even though we recruited only newly diagnosed patients and conducted blood collection as soon as possible. Second, limited by the case-control design, this study could not definitively conclude whether elevated serum TMAO was involved in the liver carcinogenesis, or if liver cancer caused the serum TMAO elevations. More prospective studies are needed to verify the causality between TMAO and PLC. Third, despite the relatively large numbers of participants in the present study, sample size may have played a role in the questionably strong associations seen in women participants, considering the much smaller female sample size. Since liver cancer is much more common in men than in women [2], it was difficult to recruit an equal number of female PLC patients in the present study. Thus, the results from women should be interpreted with caution and need to be confirmed by studies with larger female sample sizes.

## Conclusions

In conclusion, the present study revealed opposing associations between serum trimethylamine N-oxide (adverse) and its precursor choline (inverse) with the risk of PLC. We suggest that higher TMAO concentrations are associated with increased risk of PLC, while higher serum choline levels are associated with reduced risk of PLC. However, these results should be interpreted with caution and should be confirmed by large prospective studies in the future.

## Abbreviations

BMI: Body mass index
BP: Blood pressure
CI: Confidence interval
CRC: Colorectal cancer
FBG: Fasting blood glucose
FMO3: Flavin-containing monooxygenase-3
HDL-C: High-density lipoprotein cholesterol
Mets: Metabolic syndrome
NAFLD: Non-alcoholic fatty liver disease
OR: Odds ratio
PLC: Primary liver cancer
TC: total cholesterol
TG: Triglycerides
TMAO: Trimethylamine-N-oxide
WC: Waist circumference
